# Continuous Antibiotic Administration Using IRRAflow® Catheter for Treatment of Intracranial Abscess

**DOI:** 10.7759/cureus.19061

**Published:** 2021-10-26

**Authors:** Ryan M Hess, Audrey Lazar, David Smolar, Timothy E OConnor, Asham Khan, Adnan H Siddiqui, Elad I Levy

**Affiliations:** 1 Neurosurgery, University at Buffalo Neurosurgery, Buffalo, USA; 2 Neurosurgery, Middlebury College, Middlebury, USA; 3 Neurosurgery, Buffalo General Medical Center, Buffalo, USA; 4 Neurosurgery, University at Buffalo, Jacobs School of Medicine and Biomedical Sciences, Buffalo, USA

**Keywords:** intracranial abscess, irraflow, neurosurgery technology, infectious disease pathology, intrathecal antibiotics

## Abstract

Intracranial abscesses are rare lesions with an incidence of approximately 4 per 1 million people. The optimal surgical management of these lesions is still unclear. We present the case of a patient who was discovered to have an intracranial abscess after presenting with right-sided weakness. He was treated via a combination of open craniotomy and continuous antibiotic irrigation using an IRRAflow® catheter (IRRAS, Stockholm, Sweden). Use of the IRRAflow® in this fashion has not yet been described in the literature. This novel approach appears to be safe and resulted in continued decrease in the abscess burden following surgical drainage.

## Introduction

Intracranial abscesses are rare lesions with an incidence estimated at 1500-2500 cases per year in the United States [1]. Widespread use of neuroimaging has led to rapid diagnosis, decreasing the mortality rate from 40-50% to approximately 20% [2]. Treatment options include systemic antibiotic therapy, needle aspiration through a burr hole, and open craniotomy for excision [3,4].

The recently introduced IRRAflow® self-irrigating catheter system (IRRAS, Stockholm, Sweden) has been used to treat subdural hematomas and intraventricular hemorrhages [5,6] although research into other applications for this technology is still ongoing. We present the case of an intracranial abscess treated via open craniotomy and IRRAflow® placement allowing for continuous irrigation and drainage of the abscess.

## Case presentation

**Figure 1:**
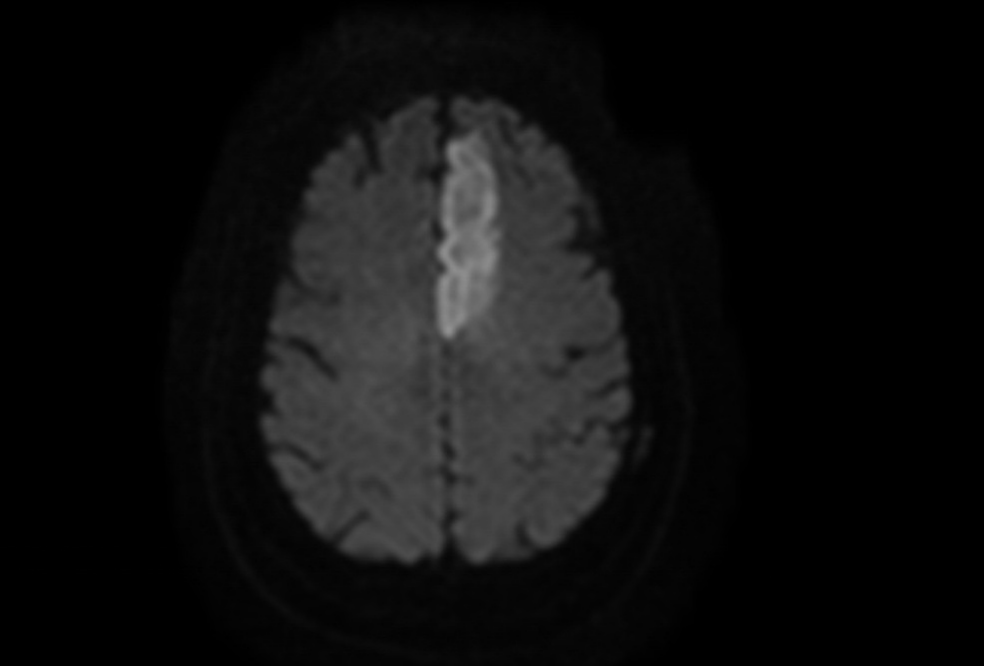
MRI DWI (left) demonstrating left ACA territory infarction. MRI, magnetic resonance imaging; ACA, anterior cerebral artery; DWI, diffusion-weighted imaging

The patient is a 63-year-old man with a medical history of hypertension, hyperlipidemia, heart failure with preserved ejection fraction, and coronary artery disease who originally presented to our center from an outside hospital with right-sided weakness and speech difficulties. On arrival, his National Institutes of Health Stroke Scale (NIHSS) score was 12 owing to right arm drift, right leg weakness, aphasia, and dysarthria. The drift was thought to be due to inability to follow commands fully. Due to lack of venous access, magnetic resonance imaging (MRI) and magnetic resonance angiography were performed instead of computed tomography angiography. A left anterior cerebral artery (ACA) infarction secondary to a left A1 occlusion was demonstrated. Diffusion-weighted imaging (DWI) and fluid-attenuated inversion recovery (FLAIR) sequences are shown in Figure 1. Given the completed infarction, no tissue plasminogen activator was administered and thrombectomy was not performed. The patient met sepsis criteria so blood cultures were obtained, and he was admitted to the neurosciences intensive care unit for further management. He was also noted to have atrial fibrillation with rapid ventricular response at that time. 

Blood cultures grew *Streptococcus constellatus* for which intravenous (IV) antibiotic therapy with ceftriaxone (2 g twice daily) and metronidazole (500 mg every 8 h) was initiated. To investigate hyperbilirubinemia on initial laboratory testing, liver ultrasonography was performed, demonstrating a hepatic abscess. He then underwent image-guided abscess drainage followed by placement of a pigtail drain. A transthoracic echocardiogram demonstrated severe mitral valve regurgitation. Given the high suspicion for endocarditis, a transesophageal echocardiogram was performed. This demonstrated mitral valve vegetations. Following completion of his medical care, the patient was deemed stable for discharge to a rehabilitation facility. The antibiotic plan at that time was 14 days of oral metronidazole (500 mg twice daily) and four weeks of IV ceftriaxone at the previous dose given his downtrending infectious markers. For stroke prevention he was placed on apixiban 5 mg twice daily. He recovered a good portion of his right-sided strength and was discharged home after completing his rehabilitation course. 

**Figure 2:**
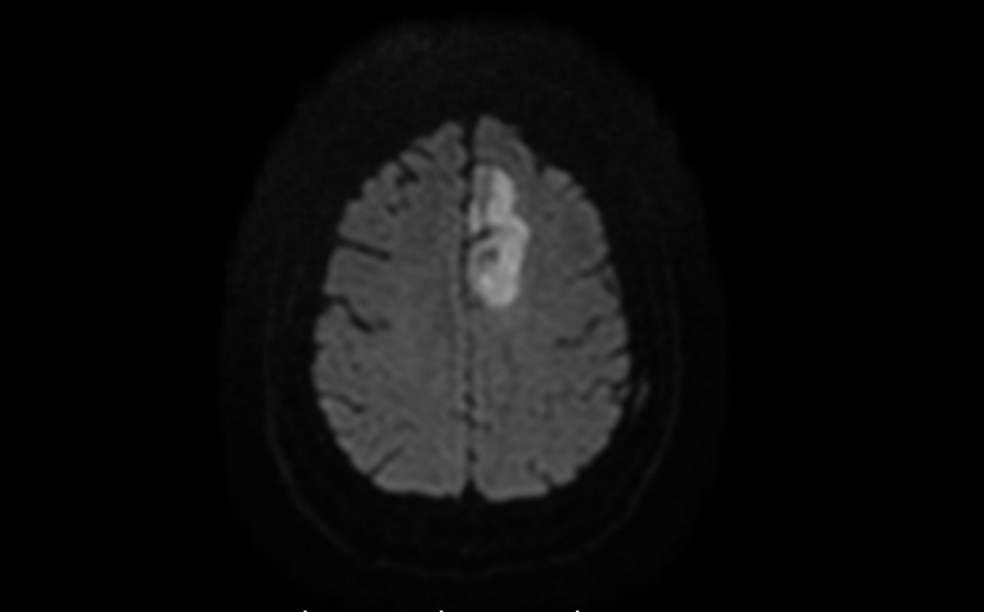
Slight increase in the area of diffusion restriction (left) in the left ACA territory. ACA, anterior cerebral artery

**Figure 3:**
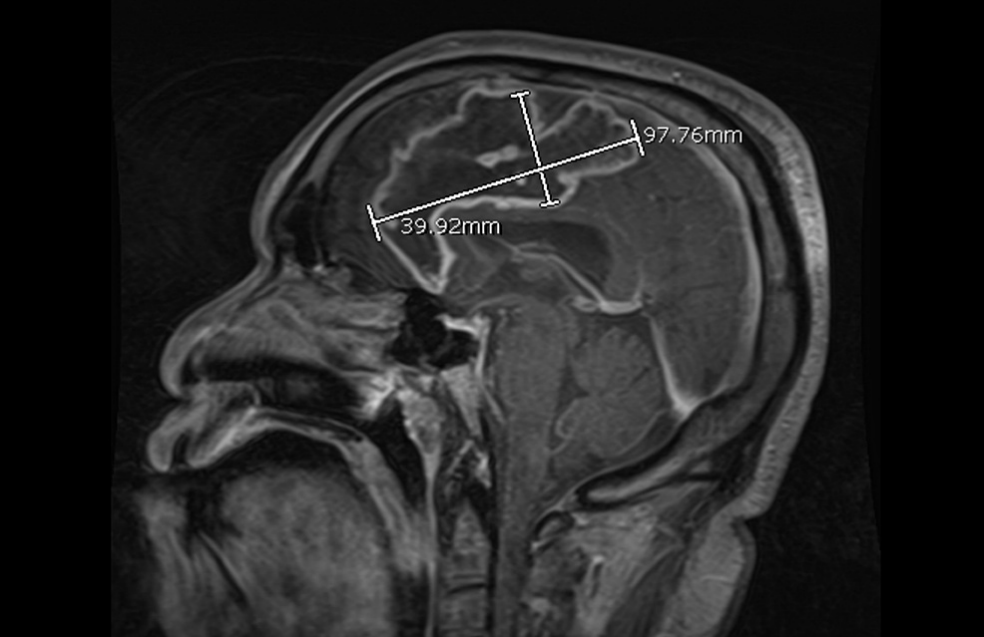
Contrast-enhanced MRI showing a 9.7 x 3.9 x 2.6 cm (98 cc) ring enhancing lesion of the left frontal lobe in the prior area of infarction. MRI, magnetic resonance imaging

The patient was readmitted to our center four days post-discharge with worsening right-sided weakness (NIHSS score of 3). Brain perfusion imaging demonstrated no new perfusion deficits or large vessel occlusions. Repeat MRI DWI/FLAIR sequences demonstrated evolution of the left ACA territory FLAIR signal and diffusion restriction (Figure 2). This was concerning for worsening ischemia or an abscess. Contrast-enhanced MRI was consistent with an intracranial abscess within the previous stroke bed (Figure 3).

 

**Figure 4:**
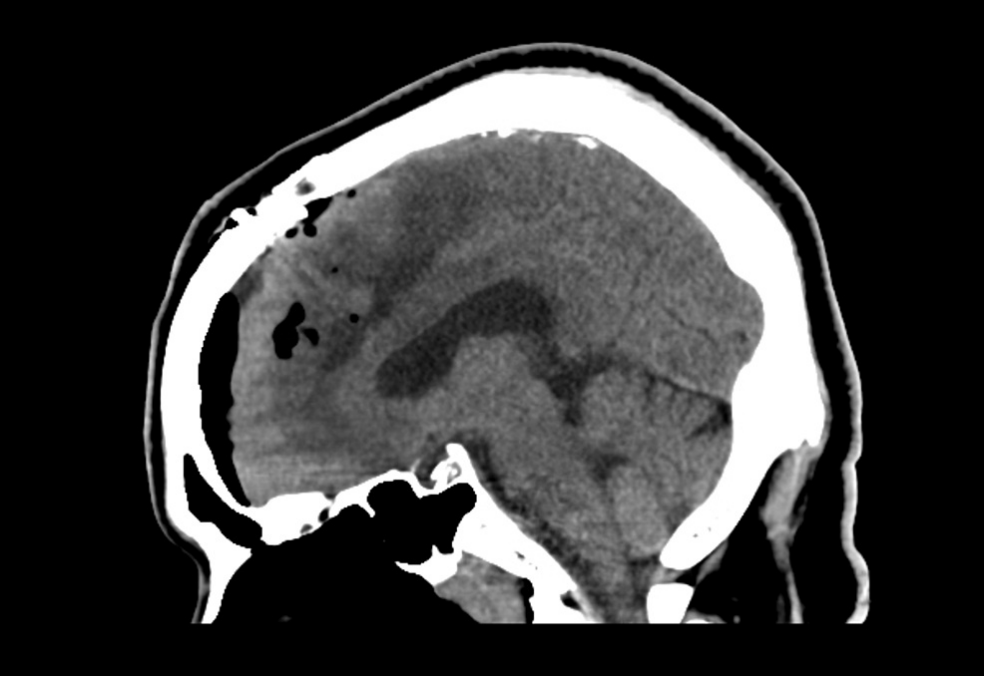
Immediate postoperative CT. CT, computed tomography

Because the abscess was abutting the frontal horn of the lateral ventricle, the patient was emergently taken to the operating room for drainage. The abscess was localized using Stealth® (Medtronic, Minneapolis, MN, USA) navigation, and an incision site was marked. After craniotomy, a small corticectomy was performed over the abscess. Thick, yellow pus was immediately encountered. Approximately 30 mL was drained; however, it was noted that there was a component of organized phlegmon. Therefore, an IRRAflow® catheter was placed in the cavity in order to irrigate and promote drainage following closure. After the procedure, imaging was obtained (Figure 4) prior to the initiation of irrigation (20 mL per hour with 0.9 normal saline). The patient was then placed on IV antibiotics consisting of vancomycin (1.75 g every 12 hours) and cefepime (2 g every 8 hours), in addition to oral metronidazole (500 mg every 6 hours) until culture results were received. On postoperative day 2, the irrigation solution was switched for 50 mg of vancomycin diluted in 1 L of normal saline, and this solution was continuously irrigated through the IRRAflow® system.

**Figure 5:**
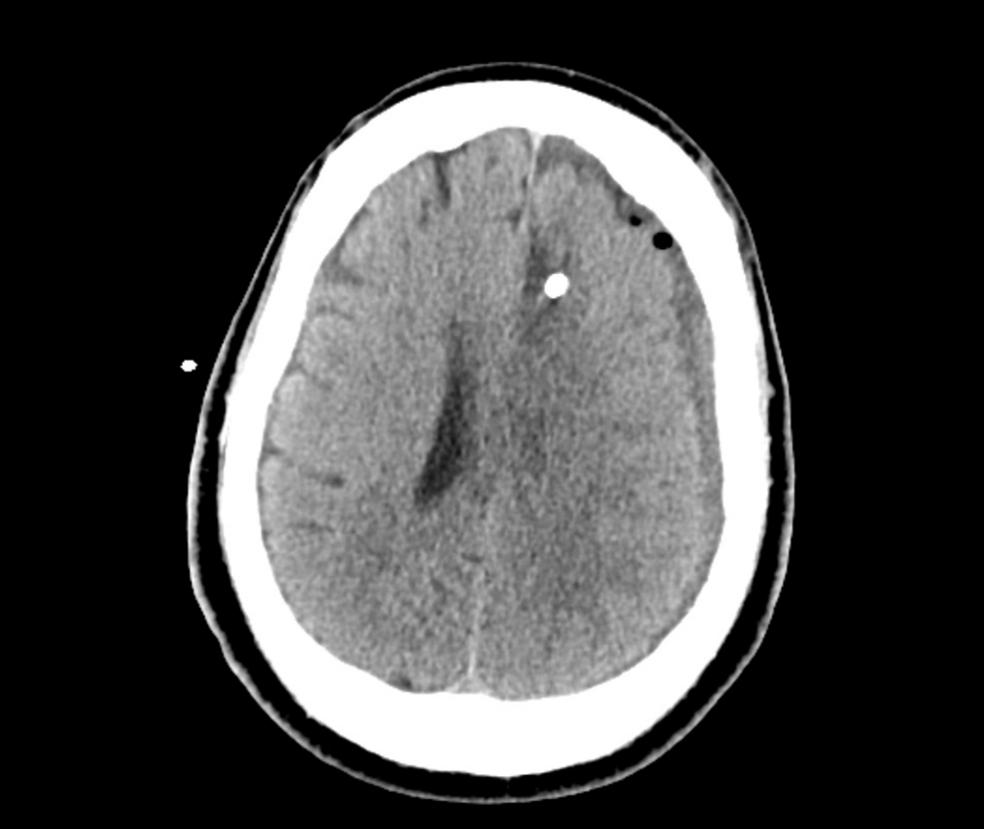
Non-contrast CT demonstrating collapsed abscess cavity and small left frontal extra-axial fluid collection. CT, computed tomography

**Figure 6:**
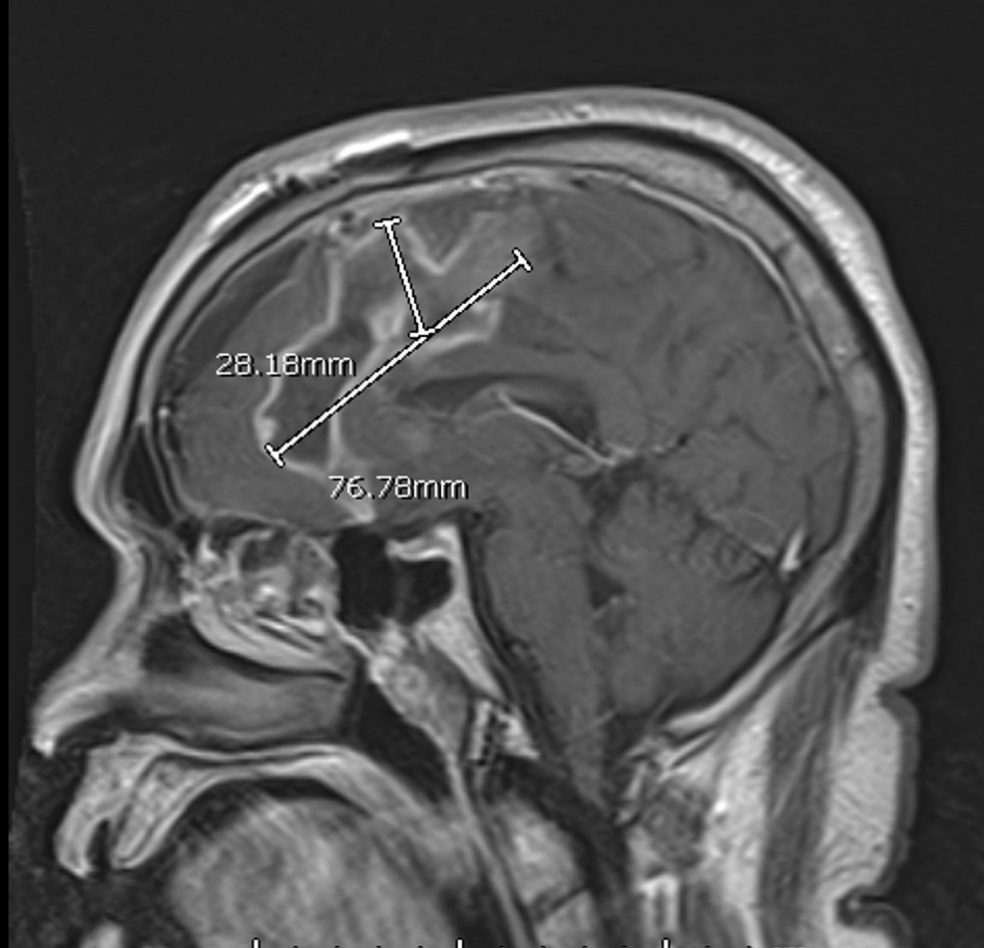
Repeat contrast-enhanced MRI demonstrating a decrease in the size of the abscess cavity. MRI, magnetic resonance imaging

After intraoperative cultures revealed *Staphylococcus hominis*, the systemic antibiotic regimen was changed to ceftriaxone (2 g daily) and metronidazole (500 mg every 8 h). The patient was observed for one week with decreasing infectious markers. Serial head CT scans were performed during this time. As demonstrated in Figure 5, a small left frontal subdural fluid collection developed. It decreased after the irrigation rate was lowered to 10 mL per hour. Repeat MRI showed significant resolution of the abscess (Figure 6). From a neurologic standpoint, the patient experienced a reduction of his NIH to 2 for improved right leg weakness, right arm drift, and aphasia. Given these findings, the drain was removed on postoperative day 8. CT obtained the following day demonstrated continued resolution of the subdural fluid collection.

The patient had an extended hospital course due to a cholecystoduodenal fistula that was found on investigation for the cause of his liver abscess. A periampullary mass thought to represent malignancy was found during endoscopic retrograde cholangiopancreatography. These issues were managed by the general surgery, oncology, and medical services. No other acute neurosurgical issues arose during the remainder of his hospitalization, and he was discharged to rehabilitation after finishing his one-month course of antibiotics. He is scheduled to follow-up in clinic following rehabilitation.

## Discussion

Multiple approaches exist for the treatment of intracranial abscesses including burr-hole aspiration, open excision, and antibiotic therapy alone [3,4]. According to retrospective case series, there is no clear consensus on optimal management options. In one retrospective study, Ratnaike et al. reported a lower mortality rate in patients undergoing burr-hole aspiration (6.6%) compared with patients undergoing open excision (12.7%) [3]. However, Tan et al. reported no difference in morbidity and mortality between the two approaches [7] . Additionally, other retrospective series comparing the two approaches demonstrated that open craniotomy performed in non-eloquent cortex was associated with decreased hospital stay, shorter duration of antibiotics, and earlier improvement in neurologic function [8,9]. As for antibiotic therapy alone, this is often reserved for patients with good neurologic condition and abscesses smaller than 2.5 cm. For abscesses 2.5 cm or larger, antibiotic therapy alone is not recommended because a 0% resolution rate has been reported [4,10].

Another factor complicating the treatment of intracranial abscesses is achieving adequate antimicrobial levels in the abscess cavity due to the significant purulence [11]. Two agents shown to have adequate penetration into abscesses are metronidazole and vancomycin. Studies have demonstrated close correlation of serum vancomycin levels with vancomycin levels in the abscess following removal [12,13]. Even with adequate antibiotic coverage, six or more weeks of antibiotic therapy is usually required [14].

To address the current issues with intracranial abscess treatment, we implemented a previously undescribed hybrid approach to treatment. Our approach allowed for direct decompression and drainage via corticectomy in addition to continuous clearance of infectious materials via the IRRAflow® system. Although vancomycin has adequate central nervous system penetration, we felt that including diluted vancomycin in our irrigation solution would directly expose the abscess cavity to an antimicrobial agent. The thought process behind this was that microbes within the abscess cavity may not be exposed to adequate levels of IV antibiotic given the thick residual purulence within the cavity. Additionally, we felt that continuous irrigation would break up the residual phlegmon and promote evacuation. 

Based on imaging, although the abscess decreased in size as a direct result of surgical drainage, it appears that the IRRAflow® allowed for further reduction (Figures 3, 4, 6) as expected given the intraoperative findings. An ideal comparison of immediate postoperative and delayed imaging is limited by technique differences. This reduction occurred somewhat rapidly and was associated with the development of an asymptomatic subdural fluid collection that made it necessary to decrease the irrigation rate. In terms of clinical improvement, the patient was noted to have improvement in the strength on his right side within the first 72 hours of treatment.

This case proved insightful in terms of learning points for further development of the treatment approach we have proposed. We chose to proceed initially with a craniotomy as we felt that stereotactic aspiration would not adequately address the lesion given its size. Similarly, we felt that corticectomy may result in shorter duration of antibiotics and more rapid improvement in neurologic function given the previously cited studies. Unfortunately, the pus was too thick to evacuate and the IRRAflow® was placed in order to aid evacuation. Had this been known before proceeding we would have opted for a burr hole and IRRAflow® placement with the help of neuronavigation. It is unclear whether a less-invasive approach would result in any difference in clinical outcome. However, in future cases a burr hole with placement of the IRRAflow® could certainly be an alternative to standard stereotactic aspiration or open evacuation. The patient in this case also developed an asymptomatic subdural fluid collection as a result of our treatment, perhaps from over-drainage. In the future, lower irrigation rates will likely be used to prevent this from occurring.

## Conclusions

In this case, IRRAflow® placement and the use of antibiotic irrigation directly into the intracranial abscess cavity was associated with further improvement in abscess size following surgical drainage. We conclude that this approach is safe and effective. Given the extremely limited data available from this study, more research is needed to further investigate the risk profile and benefits of this treatment approach compared to current treatment options. Additionally, placement of the device through burr hole might offer a more minimally invasive approach to treatment. Finally, further experience with the IRRAflow® in this setting is needed to create standardized treatment protocols.
